# Correlation of distribution characteristics and dynamic changes of gut microbiota with the efficacy of immunotherapy in *EGFR*-mutated non-small cell lung cancer

**DOI:** 10.1186/s12967-024-05135-5

**Published:** 2024-04-02

**Authors:** Wei-Chi Luo, Shi-Qi Mei, Zi-Jian Huang, Zhi-Hong Chen, Yi-Chen Zhang, Ming-Yi Yang, Jia-Qi Liu, Jing-Yan Xu, Xiao-Rong Yang, Ri-Wei Zhong, Li-Bo Tang, Lin-Xi Yin, Yu Deng, Ying-Long Peng, Chang Lu, Bao-Long Chen, Dong-Xian Ke, Hai-Yan Tu, Jin-Ji Yang, Chong-Rui Xu, Yi-Long Wu, Qing Zhou

**Affiliations:** 1 Guangdong Lung Cancer Institute, Guangdong Provincial People’s Hospital (Guangdong Academy of Medical Sciences), Southern Medical University, Guangzhou, 510080 China; 2 Guangdong Provincial Key Laboratory of Translational Medicine in Lung Cancer, Guangdong Provincial People’s Hospital (Guangdong Academy of Medical Sciences), Southern Medical University, Guangzhou, China; 3 School of Medicine, South China University of Technology, Guangzhou, China; 4 Xiamen Treatgut Biotechnology Co., Ltd, Xiamen, China; 5 Xiamen Treatgut Medical Laboratory Co., Ltd, Xiamen, China

**Keywords:** Non-small Cell Lung Cancer, Epidermal growth factor receptor, Gut microbiota, Metabolites, Antibiotics

## Abstract

**Background:**

The effects of gut microbiota and metabolites on the responses to immune checkpoint inhibitors (ICIs) in advanced epidermal growth factor receptor *(EGFR)* wild-type non-small cell lung cancer (NSCLC) have been studied. However, their effects on *EGFR*-mutated *(EGFR* +*)* NSCLC remain unknown.

**Methods:**

We prospectively recorded the clinicopathological characteristics of patients with advanced *EGFR* + NSCLC and assessed potential associations between the use of antibiotics or probiotics and immunotherapy efficacy. Fecal samples were collected at baseline, early on-treatment, response and progression status and were subjected to metagenomic next-generation sequencing and ultra-high-performance liquid chromatography-mass spectrometry analyses to assess the effects of gut microbiota and metabolites on immunotherapy efficacy.

**Results:**

The clinical data of 74 advanced *EGFR* + NSCLC patients were complete and 18 patients’ fecal samples were dynamically collected. Patients that used antibiotics had shorter progression-free survival (PFS) (mPFS, 4.8 *vs*. 6.7 months; *P* = 0.037); probiotics had no impact on PFS. Two dynamic types of gut microbiota during immunotherapy were identified: one type showed the lowest relative abundance at the response time point, whereas the other type showed the highest abundance at the response time point. Metabolomics revealed significant differences in metabolites distribution between responders and non-responders. Deoxycholic acid, glycerol, and quinolinic acid were enriched in responders, whereas L-citrulline was enriched in non-responders. There was a significant correlation between gut microbiota and metabolites.

**Conclusions:**

The use of antibiotics weakens immunotherapy efficacy in patients with advanced *EGFR* + NSCLC. The distribution characteristics and dynamic changes of gut microbiota and metabolites may indicate the efficacy of immunotherapy in advanced *EGFR* + NSCLC.

**Supplementary Information:**

The online version contains supplementary material available at 10.1186/s12967-024-05135-5.

## Background

The primary molecular subtype in driver gene-positive non-small cell lung cancer (NSCLC) involves the activation mutation of epidermal growth factor receptor *(EGFR)* [1]. *EGFR*-tyrosine kinase inhibitors (*EGFR*-TKIs) significantly prolong the overall survival (OS) of patients with advanced *EGFR*-mutated (*EGFR* +) NSCLC [2, 3]. However, *EGFR*-TKIs resistance is inevitable, and options for treatment after the onset of resistance remain limited [4–6]. In the past decade, immune checkpoint inhibitors (ICIs) have exhibited substantial clinical efficacy as standard treatment options among patients with driver gene-negative (*EGFR*-) advanced and locally advanced NSCLC [7–10]. However, the treatment outcomes differ in patients with *EGFR* + NSCLC. In a meta-analysis that included the results of five clinical trials comparing ICIs with docetaxel, ICIs significantly prolonged the OS of *EGFR*- patients but showed no benefit for *EGFR* + patients [11]. In contrast, in a phase III clinical trial (Orient-31), treatment with sintilimab plus IBI305, cisplatin, and pemetrexed was efficacious in patients with *EGFR* + NSCLC who progressed after receipt of *EGFR*-TKIs [12]. The detection of effective biomarkers to identify patients who will benefit from immunotherapy is important because of the high cost of ICIs and the characteristic immunologically ‘cold’ tumor microenvironments of *EGFR* + tumors [13]. Unfortunately, PD-L1 expression is not an appropriate choice [14, 15]. There are no existing robust biomarkers can be used for this purpose.

Multiple studies have shown that the use of antibiotics can weaken immunotherapy efficacy in NSCLC whereas the use of probiotics can strengthen it due to the resulting changes in gut microbiota [16–18]. Therefore, the gut microbiota has an important role in tumor immunotherapy. Responders exhibit high gut microbiota diversities, with increased levels of *Alistipes putredinis* and *Bifidobacterium longum*, whereas non-responders have increased levels of *Ruminococcus*_*unclassified* [19]. The gut microbiota diversity is positively correlated with the proportions of memory CD8^+^  T and natural killer cell subsets in peripheral blood; patients demonstrate prolonged progression-free survival (PFS) after the receipt of ICIs for NSCLC. *Akkermansia muciniphila* is the species most strongly associated with favorable clinical outcomes [20]. Additionally, the level of *Akkermansia muciniphila* has predictive value in patients with advanced NSCLC, regardless of whether they receive treatment with first-line or second-line ICIs [21]. The gut microbiota reportedly influences NSCLC immunotherapy in both European and Asian populations. Fecal metabolites are also considered as one of the factors that influence the efficacy of immunotherapy in NSCLC patients. Botticelli et al. revealed that fecal 2-pentanone and tridecane exhibited significant associations with early progression, whereas short chain fatty acids, lysine, and nicotinic acid were notably linked to long-term beneficial effects [22]. However, relevant studies on lung cancer have mainly focused on *EGFR-* NSCLC, and the impact of the gut microbiota and metabolites on *EGFR* + NSCLC remains unknown. Thus, it is important to elucidate the characteristics of the gut microbiota and metabolites in patients with advanced *EGFR* + NSCLC  after development of TKIs resistance, then assess its predictive value during ICIs treatment. In this study, clinical information of patients who received immunotherapy after TKIs resistance were prospectively recorded; stool samples were collected at various time points during immunotherapy for metagenomic next-generation sequencing and untargeted metabolomics based on ultra-high-performance liquid chromatography-mass spectrometry (UHPLC-MS) analyses. We aimed to characterize the gut microbiota and metabolic profiles of patients with *EGFR* + NSCLC receiving immunotherapy and identify the association between them.

## Methods

### Patients

This prospective cohort study enrolled patients with advanced *EGFR* + NSCLC who were receiving ICIs at Guangdong Provincial People’s Hospital from March 2019. The inclusion criteria were as follows: (1) Age ≥ 18 years; (2) Diagnosis of advanced *EGFR* + NSCLC; (3) Development of resistance after receipt of treatment with at least one type of TKIs; (4) Patients planned to receive  PD-1/PD-L1 inhibitor. The study was approved by the Ethics Committee at Guangdong Provincial People's Hospital, No. GDREC2019304H(R1). All patients were informed of the study and consented to the enrollment. This study was conducted in accordance with the Helsinki Declaration.

Clinicopathological characteristics including the use of antibiotics (2 months before and after the first ICIs injection) and/or probiotics (2 months before ICIs administration and/or concurrently with ICIs administration) were recorded after patient enrollment. An independent oncologist performed tumor response evaluations based on the Response Evaluation Criteria in Solid Tumors (RECIST) version 1.1. Tumor response categories include complete response (CR; complete disappearance of tumors), partial response (PR; ≥ 30% reduction in the sum of diameters compared to baseline), progressive disease (PD; ≥ 20% increase in the sum of the smallest tumor diameters), and stable disease (SD; neither CR, PR, nor PD). Patients with PR or CR were considered responders; patients with PD or SD were considered non-responders. The objective response rate (ORR) was defined as the percentage of patients achieving the best response of CR or PR. PFS was defined as the period from immunotherapy initiation until disease progression; OS was defined as the period from immunotherapy initiation until death. Patients with incomplete or missing data were excluded from the analysis of clinical features.

### Dynamically sample collection

Fecal samples were collected at four time points: baseline (before the first ICIs injection), early on-treatment (within 3 months of the first ICIs injection), response (when CR or PR had been reached), and progression status (when PD had been identified). Each sample was divided into an empty container (immediately frozen at − 80* °C*) and a container with Effcgut solution [23] stored at room temperature. Data from patients with incomplete samples were excluded from the analysis of gut microbiota.

### Fecal microbiota analysis by metagenomic next-generation sequencing

Stool DNA was extracted from approximately 0.25 g of fecal sample using the QIAamp Fast DNA Stool Mini Kit (Qiagen, Hilden, Germany) according to the manufacturer’s instructions. For quantifications of stool DNA, we used the Qubit^®^ dsDNA HS Assay kit (Thermo Fisher Scientific, MA, USA) to determine the concentration of the samples and the DNA was run on a 1.5% agarose gel to assess its quality. The Bioruptor NGS sonicator (Diagenode, BE) was used to fragment stool DNA (50–100 ng) to an average insert size of about 350 bp. DNA fragments were size selected (~ 350 bp) by VAHTSTM DNA Clean Beads according to the manufacturer's instructions. Metagenome libraries were prepared by NEBNext^®^ Ultra^™^ II DNA Library Prep Kit for Illumina according to the manufacturer’s instructions. Briefly, DNA fragments were subsequently end repaired and 3′-adenylated before Illumina adapters were added by using the NEBNext^®^ Sample Reagent Set (New England Biolabs, MA, USA). Ligation products were purified by AMPure^®^ XP beads (Beckman Coulter, CA, USA) and DNA fragments were PCR amplified with Illumina adapter-specific primers. Amplified library fragments were size selected (~ 450 bp) on a 1.5% agarose gel. Library size and quality were assessed using an HS-DNA chip on Agilent Bioanalyzer 2100 (Agilent Technologies, Palo Alto, CA), and DNA concentrations were measured by quantitative PCR with KAPA Illumina Library Quantification Kits (KAPA Biosystems, MA, USA) on ABI 7300 Plus machine (Thermo Fisher Scientific, MA, USA). After library profile analysis, each library was sequenced with 151 base-length read chemistry in a paired-end flow cell on Illumina NovaSeq platform (Illumina, Inc., San Diego, CA).

Regarding data processing, the raw data for sequencing needs to undergo quality control and de hosting processes. The software used in this step is kneadata, which combines Trimmomatic and Bowtie2. Then, the data is used for annotation analysis using Kranken2 to obtain species annotation information and corresponding relative abundance.

### Fecal metabolite analyses via UHPLC-MS

The stool samples (25 mg ± 1 mg) were taken, mixed with beads and 500 μL of extraction solution [MeOH: ACN: H_2_O, 2:2:1 (v/v)]. The extraction solution contains deuterated internal standards. The mixed solution were vortexed for 30 s. Then the mixed samples were homogenized (35 Hz, 4 min) and sonicated for 5 min in 4 ℃ water bath, the step repeat for three times. The samples were incubated for 1 h at − 40 ℃ to precipitate proteins. Then the samples were centrifuged at 12000 rpm (RCF = 13800(× g), R = 8.6 cm) for 15 min at 4 ℃. The supernatant was transferred to a fresh glass vial for analysis. The quality control (QC) sample was prepared by mixing an equal aliquot of the supernatant of samples.

MS analyses were performed using an UHPLC system (Vanquish, Thermo Fisher Scientific) with a Waters ACQUITY UPLC BEH Amide (2.1 × 50 mm, 1.7 μm) coupled to Orbitrap Exploris 120 mass spectrometer (Orbitrap MS, Thermo). The mobile phase consisted of 25 mmol/L ammonium acetate and 25 mmol/L ammonia hydroxide in water (pH = 9.75) (A) and acetonitrile (B). The auto-sampler temperature was 4 ℃, and the injection volume was 2 μL. The Orbitrap Exploris 120 mass spectrometer was used for its ability to acquire MS/MS spectra on information-dependent acquisition (IDA) mode in the control of the acquisition software (Xcalibur, Thermo). In this mode, the acquisition software continuously evaluates the full scan MS spectrum. The ESI source conditions were set as following: sheath gas flow rate as 50 Arb, Aux gas flow rate as 15 Arb, capillary temperature 320 ℃, full MS resolution as 60000, MS/MS resolution as 15000, collision energy: SNCE 20/30/40, spray voltage as 3.8 kV (positive) or − 3.4 kV (negative), respectively.

Regarding data processing, the raw data were converted to the mzXML format using ProteoWizard and processed with an in-house program, which was developed using R and based on XCMS, for peak detection, extraction, alignment, and integration. Then an in-house MS2 database was applied in metabolite annotation. The cutoff for annotation was set at 0.3. Then, the missing values were filled up by half of the minimum value. Also, internal standard normalization method was employed in this data analysis. The final dataset containing the information of peak number, sample name and normalized peak area was imported to SIMCA16.0.2 software package (Sartorius Stedim Data Analytics AB, Umea, Sweden) for multivariate analysis.

### Statistical analysis

All statistical analyses were conducted using R statistical software (R version 4.2.3, http://www.R-project.org/.). For clinical characteristics, we performed comparisons between the antibiotic and no antibiotic (or probiotic and no probiotic) cohorts using the Chi-squared or Fisher’s exact test for categorical variables and Student’s *t* or Mann–Whitney *U* test for continuous variables, as appropriate. After Kaplan–Meier analyses, we used log-rank test to compare survival times. We selected important variables for multivariate analyses. Microbiota alpha diversity analysis and principal components analysis were conducted using the ‘vegan’ package. We performed various analyses to identify differences in taxa, metabolites, and abundance among groups. We used the corr.test function in the R package ‘psych’ to assess correlations between microbial taxa and metabolites. KEGG (http://www.genome.jp/kegg/) were used for pathway enrichment analysis. All tests were two-sided, and the threshold for statistical significance was regarded as *P* < 0.05.

## Results

### Patient characteristics

Between March 2019 and September 2022, 74 advanced *EGFR* + NSCLC patients were enrolled after applied exclusion criteria (Additional file 1: Fig S1). Among those patients, 13 (13/74, 17.6%) received antibiotics and 11 (11/74, 14.9%) received probiotics (Bio-Three Tablets; Live Combined Bifidobacterium and Lactobacillus Tablets) (Table 1). The median age was 56 years (range: 29–74 years), and two-thirds of the patients were women. Most patients were non-smokers (57/74, 77%). Additionally, most patients had been diagnosed with non-squamous cell carcinoma (69/74, 93.2%), and 63.5% were PD-L1 positive (PD-L1 ≥ 50%, 26/74, 35.1%; 1–49%, 21/74, 28.4%). Before receipt of ICIs, half of the patients had liver or brain metastases, and 95.9% (71/74) had performance status (PS) scores ≤ 1. More than half of the patients (42/74, 56.8%) received immunotherapy beyond the third line, and most patients (67/74, 90.5%) received combination immunotherapy. Baseline factors were comparable between patients in the antibiotic and no antibiotic groups, as well as between patients in the probiotic and no probiotic groups.

**Table 1:** Baseline demographic characteristics of all participants

	Probiotics			Antibiotics		
	No (N = 63)	Yes (N = 11)	*p* value	No (N = 61)	Yes (N = 13)	*p* value
Gender						
Female	39 (61.9%)	7 (63.6%)	1.000	41 (67.2%)	5 (38.5%)	0.065
Male	24 (38.1%)	4 (36.4%)		20 (32.8%)	8 (61.5%)	
Age,y						
Mean (SD)	54.3 (10.0)	58.2 (8.57)	0.202	54.7 (9.45)	55.9 (11.9)	0.734
Median [Min, Max]	56.0 [29.0, 74.0]	59.0 [47.0, 69.0]		55.0 [34.0, 74.0]	58.0 [29.0, 70.0]	
Smoking						
No	47 (74.6%)	10 (90.9%)	0.439	48 (78.7%)	9 (69.2%)	0.480
Yes	16 (25.4%)	1 (9.1%)		13 (21.3%)	4 (30.8%)	
ECOG PS^a^						
> 1	2 (3.2%)	1 (9.1%)	0.387	2 (3.3%)	1 (7.7%)	0.445
≤ 1	61 (96.8%)	10 (90.9%)		59 (96.7%)	12 (92.3%)	
Histology						
Non-SCC^b^	59 (93.7%)	10 (90.9%)	1.000	56 (91.8%)	13 (100%)	1.000
SCC	3 (4.8%)	0 (0%)		3 (4.9%)	0 (0%)	
Missing	1 (1.6%)	1 (9.1%)		2 (3.3%)	0 (0%)	
Liver or brain metastases						
No	28 (44.4%)	6 (54.5%)	0.770	27 (44.3%)	7 (53.8%)	0.747
Yes	35 (55.6%)	5 (45.5%)		34 (55.7%)	6 (46.2%)	
PD-L1						
< 1%	12 (19.0%)	3 (27.3%)	0.243	14 (23.0%)	1 (7.7%)	0.408
≥ 50%	20 (31.7%)	6 (54.5%)		21 (34.4%)	5 (38.5%)	
1–49%	20 (31.7%)	1 (9.1%)		16 (26.2%)	5 (38.5%)	
NA^c^	11 (17.5%)	1 (9.1%)		10 (16.4%)	2 (15.4%)	
Treatment line						
> 3	37 (58.7%)	5 (45.5%)	0.515	34 (55.7%)	8 (61.5%)	0.766
≤ 3	26 (41.3%)	6 (54.5%)		27 (44.3%)	5 (38.5%)	
Treatment regimen						
ICIs^d^	7 (11.1%)	0 (0%)	0.121	6 (9.8%)	1 (7.7%)	0.812
ICIs + Chemo^e^/Ais^f^	29 (46.0%)	9 (81.8%)		30 (49.2%)	8 (61.5%)	
ICIs + Chemo + Ais	27 (42.9%)	2 (18.2%)		25 (41.0%)	4 (30.8%)	

^a^Eastern Cooperative Oncology Group performance status

^b^Squamous cell carcinoma

^c^Non applicable

^d^Immune checkpoint inhibitors

^e^Chemotherapy

^f^Anti-angiogenesis

### Antibiotic use was correlated with immunotherapy efficacy for *EGFR* + NSCLC

**Fig. 1:**
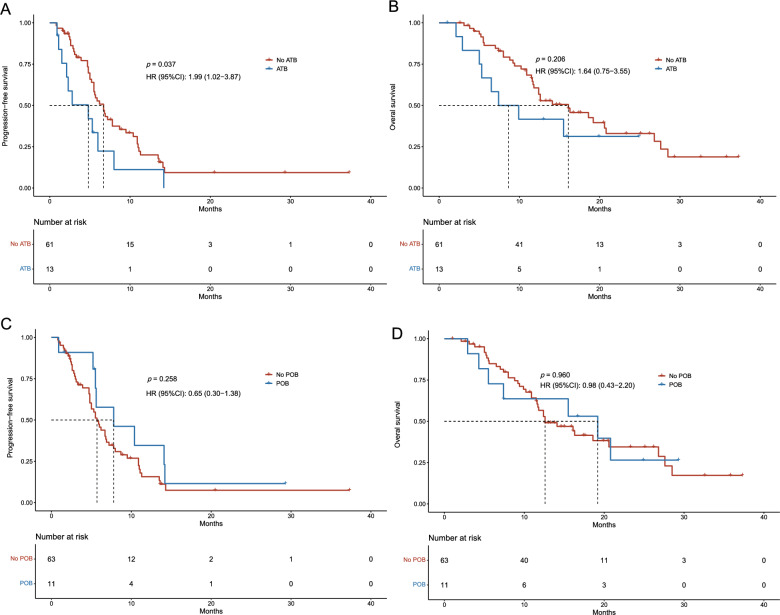
Survival outcomes for patients with advanced *EGFR*-mutated (*EGFR* +) NSCLC. **A** Progression-free survival (PFS) in patients with and without ATB use. **B** Overall survival (OS) in patients with and without ATB use. **C** PFS in patients with and without POB use. **D** OS in patients with and without POB use. *ATB* antibiotic, *POB* probiotic.

Compared with patients in the no antibiotic group, patients who received antibiotics had a shorter PFS (*P* = 0.037) (Fig. 1A). The ORR (*P* = 0.800) (Additional file 1: Fig S2A) and OS (*P* = 0.206) were similar between both groups (Fig. 1B). The ORR (*P* = 0.742), PFS (*P* = 0.258), and OS (*P* = 0.960) were similar between patients who did and did not receive probiotics (Fig. 1C, D, and Additional file 1: Fig S2B).

Univariate Cox regression analyses showed that antibiotic use was significantly associated with worse PFS (*P* = 0.042), whereas probiotic use had no impact on PFS (Additional file 2: Table S1). OS was unaffected by the use of antibiotics or probiotics (Additional file 2: Table S2). In multivariable analysis, antibiotic use was identified as an independent prognostic factor for PFS (mean PFS, 4.8 *vs*. 6.7 months; hazard ratio [HR], 3.18; 95% confidence interval [CI] 1.48–6.85; *P* = 0.003) and OS (mean OS, 7.4 *vs*. 16.1 months; HR, 3.64; 95% CI 1.30–10.17; *P* = 0.014). Other independent factors influencing PFS included histology type, PD-L1 expression level, treatment lines, and treatment regimen (*P* < 0.05). Factors influencing OS included the PS score, presence of liver or brain metastasis, histology type, and treatment lines (*P* < 0.05).

**Fig. 2:**
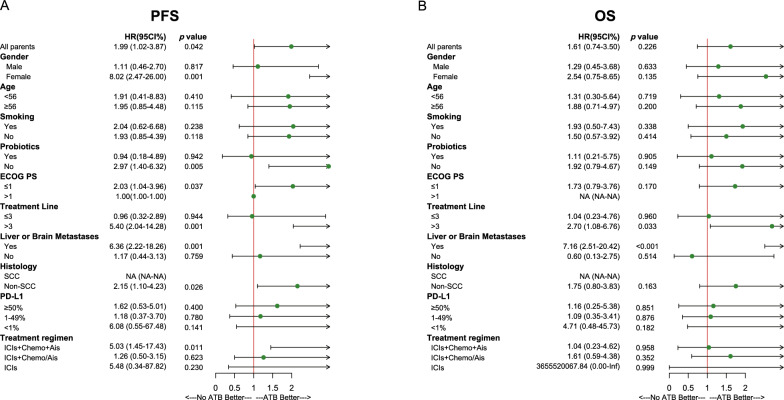
Subgroup analyses of baseline characteristics and their impact on patient survival. **A** Forest plot of impact on progression-free survival (PFS). **B** Forest plot of impact on overall survival (OS). *ECOG PS* Eastern Cooperative Oncology Group performance status, *SCC* squamous cell carcinoma, *ICIs* immune checkpoint inhibitors, *Chemo* chemotherapy, *Ais *Anti-angiogenesis, *NA* non applicable, *Inf* infinite, *ATB* antibiotic.

Subgroup analyses also revealed the impacts of antibiotic and probiotic use on PFS and OS. The use of antibiotics was significantly associated with worse PFS in women, non-users of probiotic, patients with PS scores ≤ 1, patients receiving ICIs beyond the third line, patients with liver or brain metastases, patients with non-squamous cell carcinoma, and patients treated with immunotherapy plus chemotherapy and  anti-angiogenesis (Ais) therapy (*P* < 0.05; Fig. 2A). In the subgroup of patients with liver or brain metastases and patients receiving ICIs beyond the third line, antibiotic use was significantly associated with poor OS (*P* < 0.05) (Fig. 2B). However, probiotic use had no impact on PFS or OS (Additional file 1: Fig S3).

### Gut microbiota was associated with immunotherapy response in patients with *EGFR* + NSCLC

**Fig. 3:**
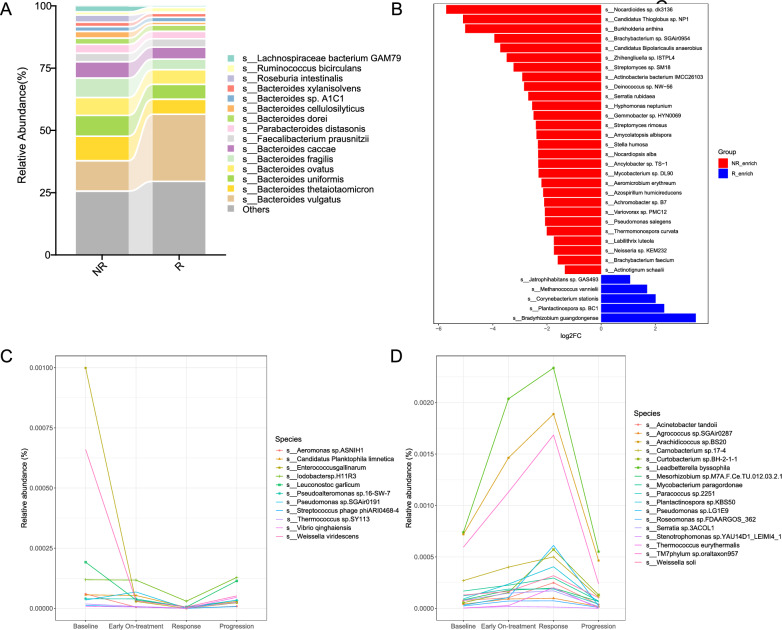
Gut microbiota composition in patients with advanced *EGFR* + NSCLC. **A** Top 15 gut microbiota components at the species level in R and NR. **B** Some of differentially abundant gut microbiota between R and NR. **C** Microbiota species belonging to the U type. **D** Microbiota species belonging to the inverted U type. *R* responder, *NR* non-responders

Baseline clinicopathological factors were comparable between responders and non-responders (Additional file 2: Table S3). To explore the landscape of gut microbiota and its association with ICIs efficacy among patients with *EGFR* + NSCLC, we conducted metagenomic next-generation sequencing experiments using fecal samples. The baseline alpha and beta diversities of the gut microbiota in responders and non-responders were similar (Additional file 1: Figs S4, S5). The relative abundances of gut microbiota components were calculated at the phylum, class, order, family, genus, and species levels in both groups. At the species level, the top 15 gut microbiota included *Bacteroides vulgatus*, *Bacteroides thetaiotaomicron*, *Bacteroides uniformis*, and others (Fig. 3A). There were 159 differentially abundant species between responders and non-responders. Some of microbiota were shown in Fig. 3B. The gut microbiota of responders was predominantly enriched in *Bradyrhizobium guangdongense*, *Plantactinospora* sp. BC1, *Corynebacterium stationis*, and *Methanococcus vannielii* (*P* < 0.05); the gut microbiota enriched in non-responders were mainly *Nocardioides* sp. dk3136, *Candidatus thioglobus* sp. NP1, *Burkholderia anthina*, and *Brachybacterium* sp. SGAir0954 (*P* < 0.05). However, we did not find  the presence of gut microbiota in patients with advanced *EGFR* + NSCLC that have been associated with effective immunotherapy in *EGFR*- NSCLC patients, such as *Akkermansia muciniphila*, *Bifidobacterium bifidum*, or *Bifidobacterium breve*.

We conducted serial dynamical fecal sample analyses of 18 patients at four time points: baseline, early on-treatment, response, and progression status. Two dynamic types were identified. The first type (U type) exhibited a decrease in relative abundance from baseline to response, followed by an increase from response to progression. This type was characterized by *Enterococcus gallinarum*, *Iodobacter* sp. H11R3, *Pseudomonas* sp. SGAir0191, and *Vibrio qinghaiensis*, among others (Fig. 3C). The other type (inverted U type) showed an increase in relative abundance from baseline to response, followed by a decrease from response to progression. This type was characterized by *Mycobacterium paragordonae*, *Thermococcus eurythermalis*, *Stenotrophomonas* sp. YAU14D1_LEIMI4_1, and other bacteria (Fig. 3D).

### Gut metabolites were associated with immunotherapy response in patients with *EGFR* + NSCLC

**Fig. 4:**
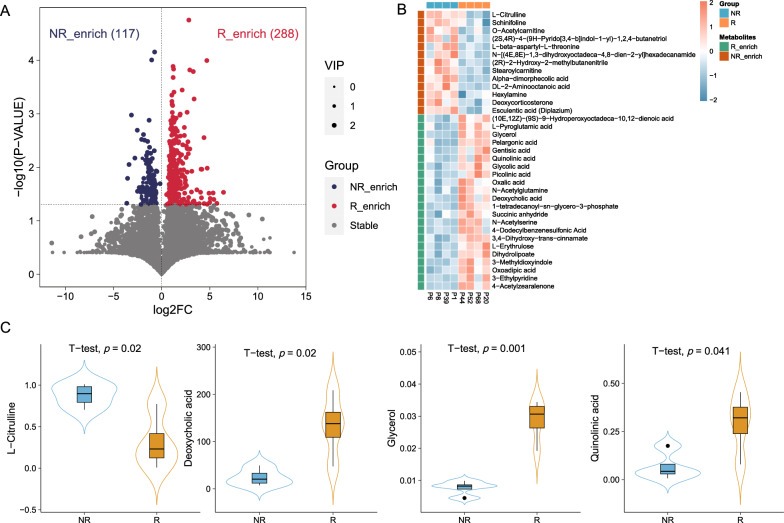
Metabolite distributions in patients with advanced *EGFR* + NSCLC. **A** Volcano plot showing differentially expressed metabolites between R and NR. **B** Heatmap of 35 annotated differentially expressed metabolites in R and NR at baseline. **C** Comparisons of L − citrulline, deoxycholic acid, glycerol, and quinolinic acid levels between R and NR

To further explore the effects of fecal metabolites on immunotherapy efficacy in patients with advanced *EGFR* + NSCLC, untargeted metabolomics revealed differences in metabolic profile between responders and non-responders. At the super class level, these metabolites included lipids and lipid-like molecules, as well as organic acids and derivatives, among others. 405 differentially expressed metabolites were identified between responders and non-responders, 35 of which were annotated (Fig. 4A). Among these, 22 metabolites were upregulated in responders, including deoxycholic acid (*P* = 0.02), glycerol (*P* = 0.001), and quinolinic acid (*P* = 0.041); 13 metabolites were upregulated in non-responders, including L-citrulline (*P* = 0.02) (Fig. 4B, C).

**Fig. 5:**
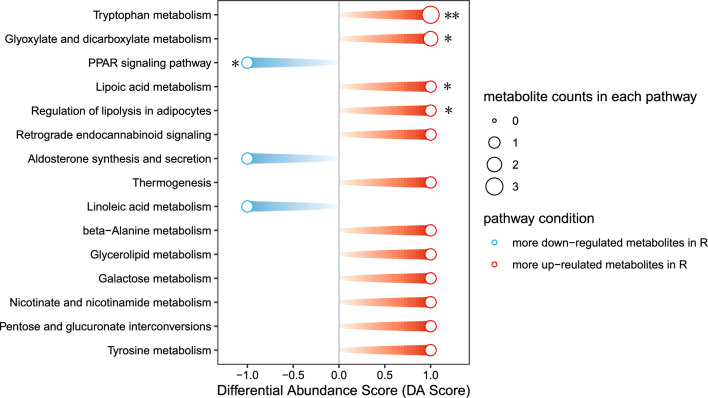
Metabolic pathways analyzed between R and NR. *P < 0.05, **P <0.01

We analyzed the metabolic pathways enriched among differentially expressed metabolites, and found that five metabolic pathways were significantly enriched. The differentially expressed metabolites in responders were enriched in the tryptophan metabolism, glyoxylate and dicarboxylate metabolism, lipoic acid metabolism, and regulation of lipolysis in adipocytes, while the differentially expressed metabolites in non-responders were enriched in the PPAR signaling pathway. (Fig. 5).

### Gut microbiota and metabolites associations

**Fig. 6:**
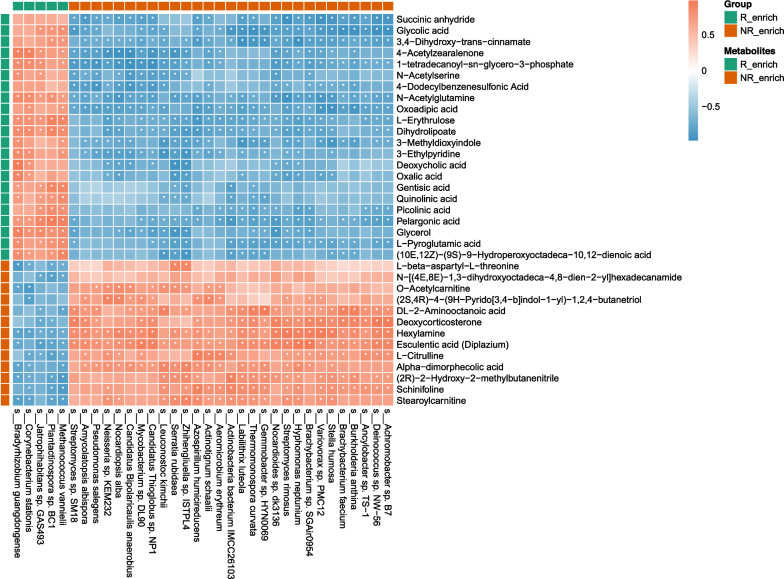
Spearman correlations between microbiota and metabolites based on differential analysis. *P < 0.05

To explore potential mechanisms underlying the effects of gut microbiota and fecal metabolites on immunotherapy efficacy in patients with advanced *EGFR* + NSCLC, correlation analysis between differentially abundant gut microbiota and metabolites at baseline in responders and non-responders showed significant associations (Fig. 6). Gut microbiota enriched in responders were positively correlated with metabolites abundant in responders and negatively correlated with metabolites abundant in non-responders. We identified metabolites like glycerol, deoxycholic acids, and quinolinic acid that could potentially influence the efficacy of anti-tumor immunotherapy. These metabolites showed positive correlations with *Bradyrhizobium guangdongense* (*P* < 0.05), as well as negative correlations with *Serratia rubidaea* and *Zhihengliuella* sp. ISTPL4 (*P* < 0.05). Similarly, the gut microbiota abundant in non-responders showed positive correlations with metabolites abundant in non-responders and negative correlations with metabolites abundant in responders. For example, L−citrulline was enriched in non-responders; it exhibited positive correlations with *Brachybacterium faecium* and *Ancylobacter* sp. TS − 1 (*P* < 0.05), as well as negative correlations with *Corynebacterium stationis*, *Jatrophihabitans* sp. GAS493, *Plantactinospora* sp. BC1, and *Methanococcus vannielii* (*P* < 0.05).

## Discussion

To our knowledge, this is the first multi-omics study to explore correlations between the use of antibiotics or probiotics, distribution characteristics and dynamic changes of gut microbiota, fecal metabolites, and immunotherapy efficacy among patients with advanced *EGFR* + NSCLC. Our results show that antibiotic use weakened immunotherapy efficacy in these patients, whereas probiotic use had no impact on treatment efficacy. Two dynamic types were identified in analyzing gut microbiota at baseline, early on-treatment, response and progression status. In the U type, relative abundances of gut microbiota decreased from baseline to response, then increased from response to progression; in the inverted U type, relative abundances of gut microbiota increased from baseline to response, then decreased from response to progression. Metabolites may serve as predictors of immunotherapy efficacy in advanced *EGFR* + NSCLC. There were significant correlations between gut microbiota and metabolites in our cohort. To our knowledge, this is the first investigation of microbiota and metabolic factors influencing immunotherapy efficacy in patients with advanced *EGFR* + NSCLC.

The present study is the first to show that the use of antibiotics significantly weakens immunotherapy efficacy in *EGFR* + NSCLC. Previous studies indicated that the use of antibiotics, which affects the composition and diversity of gut microbiota, was associated with poor immunotherapy outcomes in patients with advanced *EGFR-* NSCLC, both in European and Asian populations [24–26]. Fidelle et al. reported [27] that the use of antibiotics led to the colonization of *Enterocloster*, resulting in the down-regulation of mucosal addressin cell adhesion molecule 1. That facilitated the migration of α4β7^+^  CD4^+^ Treg17 cells to the tumor, mediating immune evasion and compromising the efficacy of immunotherapy. Additionally, Takada et al. found that oral administration of the probiotic *Clostridium butyricum* was associated with favorable clinical outcomes among patients with advanced or recurrent NSCLC who received anti-PD-1 monotherapy [28]. Furthermore, preclinical studies have revealed that *Clostridium butyricum* treatment of colorectal cancer cells result in MYC degradation through increased proteasome-mediated ubiquitination, thereby enhancing the anti-PD-1 immunotherapeutic efficacy [29]. However, the use of probiotics had no impact on immunotherapy efficacy among patients with advanced *EGFR* + NSCLC in our study. This discrepancy might be related to differences in factors such as medication cycle or the types or dosages of probiotics, ethnic factors, dietary habits, or other factors.

We did not identify a significant predictive effect of gut microbiota alpha or beta diversities with respect to immunotherapy efficacy among patients with *EGFR* + NSCLC. The results of some studies have suggested that the absence of immunotherapy benefits in these patients may be related to their characteristic “cold” tumor microenvironments [13, 30]. Consequently, compared with advanced *EGFR-* NSCLC, the alpha and beta diversities may be lower at baseline in *EGFR* + NSCLC, contributing to the absence of differences in diversity between responders and non-responders. We are currently conducting a comparative analysis of the differences in diversity between these two molecular subtypes of NSCLC. Significant differences were observed in gut microbiota between responders and non-responders, represented by *Bradyrhizobium guangdongense* and *Nocardioides* sp. dk3136, which differed from published reports concerning *EGFR-* NSCLC. Previous studies have reported that gut microbiota affects the efficacy of immunotherapy in *EGFR-* NSCLC [20, 21, 31, 32], including *Akkermansia muciniphila*, *Bifidobacterium bifidum*, and *Bifidobacterium breve*. Routy et al. demonstrated that *Akkermansia muciniphila* enhances immune checkpoint inhibitor efficacy by recruiting CCR9^+^ + CXCR3^+^  CD4^+^  T cells in an IL-12-dependent manner [20]. Their subsequent larger-scale prospective study confirmed its predictive value for immunotherapy response in advanced NSCLC patients [21]. However, in our study, we did not find that gut microbiota affects the efficacy of immunotherapy. These findings suggest that patients with *EGFR* + NSCLC have a distinct gut microbiota profile relative to patients with *EGFR*- NSCLC, and our findings provide a basis for future advances in live bacterial therapeutics.

Four time points capture key changes in the gut microbiota. The baseline time point represents the state of the gut microbiota prior to immunotherapy, whereas the early on-treatment time point corresponds to a short period after immunotherapy begins. The response status reflects a stage when the tumor exhibits a positive response after a duration of immunotherapy, and the progression status indicates a long period of intervention. To a certain extent, these four time points can encapsulate the entire process of immunotherapy. And these four time points are closely related to the efficacy of immunotherapy. By concentrating on both the optimal tumor response and disease progression, which embody two extremes of treatment outcomes, we are more likely to detect crucial alterations in the gut microbiota. Through dynamic analysis, we have identified two dynamic types including U type and inverted U type during immunotherapy. Jin et al. observed stable gut microbiota during nivolumab treatment in patients with advanced NSCLC [19]. The results of another observational study suggested that microbes such as *Lachnospiraceae*, *Ruminococcaceae*, and butyrate-producing bacteria could serve as predictors of favorable ICIs responses; researchers observed that the abundances of these microbiota tended to decrease during development of secondary resistance [33]. Differences in study methodology may explain these discrepancies between studies. Unlike studies involving 16S rRNA sequencing, we used metagenomic next-generation sequencing, which can more readily characterize multiple species. Differences in experimental design may also have contributed to the observed discrepancies. Jin et al. conducted a dynamic analysis using longitudinal samples at various time points, regardless of therapeutic efficacy. Zeng et al. only collected fecal samples at three time points: baseline, primary response, and resistance; additionally, they only observed changes in gut microbiota at the time of resistance confirmation [33]. In contrast, we selected four time points (baseline, early on-treatment, response, and resistance status), which may have allowed us to identify key changes in microbiota dynamics. The U and inverted U types of gut microbiota dynamics identified in our study suggest an association between the development of response or resistance status and specific intestinal microbiota components that could serve as predictors for distinct functional responses to immunotherapy. Based on these findings, we suspect that the manipulation of gut microbiota patterns could facilitate achievement of long-term responses and delay ICIs resistance. However, further validation studies are needed to confirm our findings.

There is evidence of close associations between metabolites and tumors. Glycerol induces apoptosis in A549 cells by increasing the expression of BAX, CASP ASE-3, CASP ASE-9, TIMP-1, TIMP-2 expression and decreasing the expression of BCL-2 [34]. Additionally, quinolinic acid exhibits cytotoxic effects on B16 F10 and RAW 264.7 cells  [35]. Our research has revealed the differences in abundance of glycerol and quinolinic acid between responders and non-responders, yet further investigation is needed to understand the correlation between these metabolites with immunotherapy efficacy in patients with advanced EGFR+ NSCLC.

Gut microbiota possess the ability to secrete metabolites, potentially influencing immunotherapy outcomes. Zhu et al. found that butyrate secreted by intestinal microbiota enhances the efficacy of immunotherapy by modulating CD8^+^  T cell TCR signaling [36]. Owing to population heterogeneity, the gut microbiota profiles identified in our study differ from those previously reported in wild-type NSCLC patients, and there are no short-chain fatty acids in the differences in fecal metabolites. However, there are still some metabolites that have correlation with tumor, including deoxycholic acid, glycerol, and quinolinic acid. Those metabolites and those enriched in metabolic pathways are of importance. We believe that the correlation between these metabolites and the intestinal microbiota will serve as a foundation for deeper mechanistic studies.

This study had some limitations. First, it was a single-center observational clinical study with a relatively small sample size. However, we selected a homogeneous population of individuals with *EGFR* + NSCLC and collected fecal samples from four time points. Second, although we identified correlations between the gut microbiota composition and fecal metabolites, further in vivo and in vitro validation experiments are needed.

## Conclusion

The use of antibiotic can weaken immunotherapy efficacy in patients with advanced *EGFR* + NSCLC, whereas probiotic use does not have an impact on efficacy. The distribution characteristics and dynamic changes of gut microbiota and metabolites may indicate the efficacy of immunotherapy in advanced *EGFR* + NSCLC. This study represents the first comprehensive exploration of microbiota and metabolic factors influencing immunotherapy efficacy in patients with advanced *EGFR* + NSCLC. It will also provide insights for the development of novel therapeutic strategies.

### Supplementary Information

**Additional file 1: Figure S1.** Study flowchart. **Figure S2.** Effects of ATB and POB treatments on ICIs responses. (A) With and without ATB treatment. (B) With and without POB treatment. ATB, antibiotic; POB, probiotic. **Figure S3.** Forest plot of subgroup analysis according to baseline characteristics for progression-free survival (PFS) (A) and overall survival (OS) (B) in all included patients. ECOG PS, Eastern Cooperative Oncology Group performance status; SCC, squamous cell carcinoma; ICIs, immune checkpoint inhibitors; Chemo, chemotherapy; Ais, Anti-angiogenesis therapy; NA, non applicable; Inf, infinite; POB, probiotic. **Figure S4.** Comparison of gut microbiota alpha diversity between R and NR. R, responder; NR, non-responders. **Figure S5.** Comparison of gut microbiota beta diversity between R and NR. R, responder; NR, non-responders.

**Additional file 2: Table S1.** Univariate and Multivariate Analysis of Clinical parameters on PFS. **Table S2.** Univariate and Multivariate Analysis of Clinical parameters on OS. **Table S3.** Baseline demographic characteristics between R and NR groups

## Data Availability

Data are available upon reasonable request. All data relevant to the study are included in the article or uploaded as Additional file information.

## Abbreviations

ICIs: Immune checkpoint inhibitors
EGFR: Epidermal growth factor receptor
EGFR +: EGFR-mutated
EGFR-: Driver gene-negative
TKIs: Tyrosine kinase inhibitors
NSCLC: Non-small cell lung cancer
PFS: Progression-free survival
OS: Overall survival
UHPLC-MS: Ultra-high-performance liquid chromatography-mass spectrometry
RECIST: Response Evaluation Criteria in Solid Tumors
CR: Complete response
PR: Partial response
PD: Progressive disease
SD: Stable disease
ORR: Objective response rate
HR: Hazard ratio
CI: Confidence interval
Ais: Anti-angiogenesis
