# Supplementary figures and images for: Correlation of distribution characteristics and dynamic changes of gut microbiota with the efficacy of immunotherapy in EGFR-mutated non-small cell lung cancer
Source: J Transl Med. 2024 Apr 2;22:326. doi: 10.1186/s12967-024-05135-5 (PMC10985957; doi:10.1186/s12967-024-05135-5)

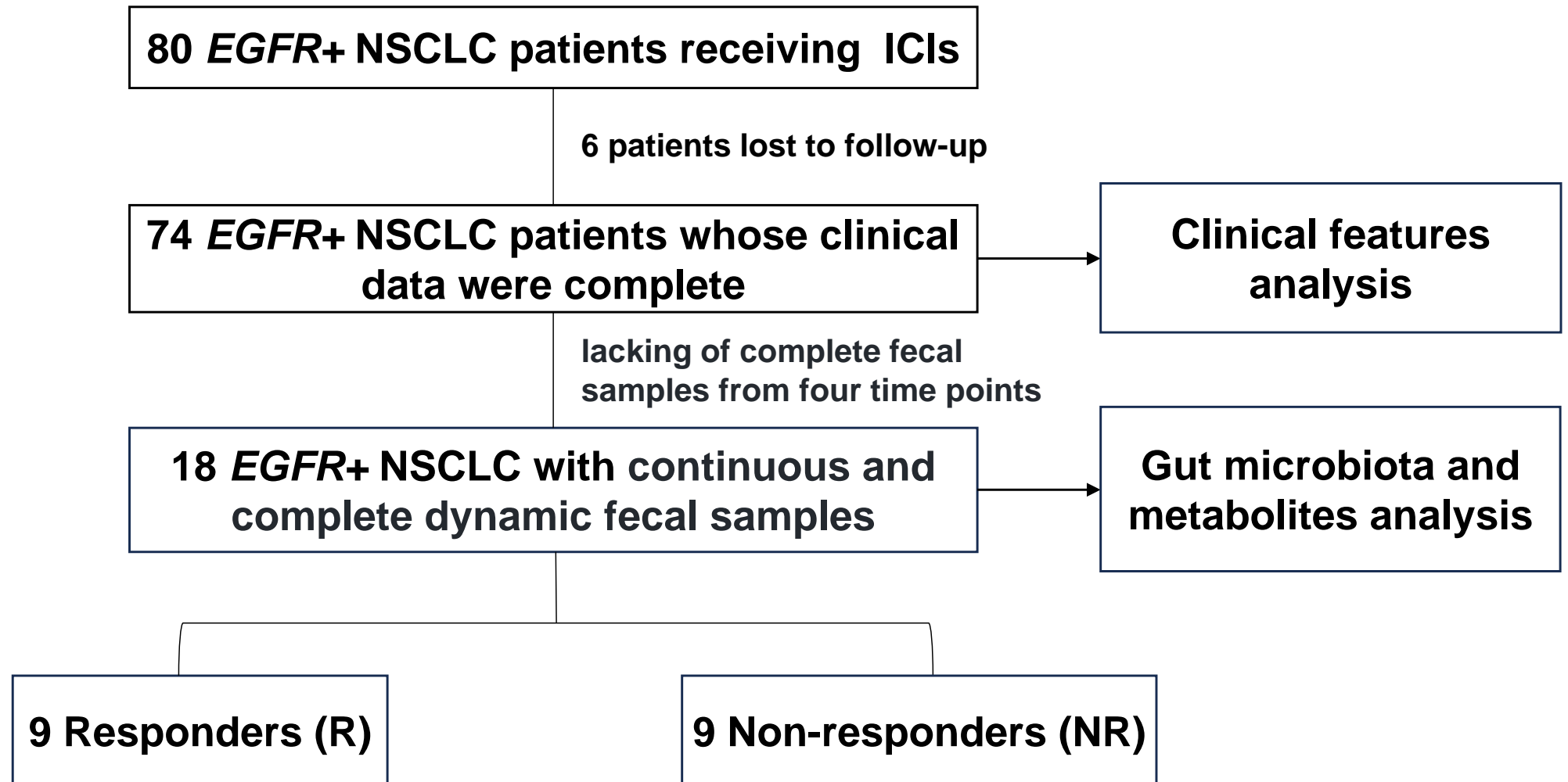

SFig2

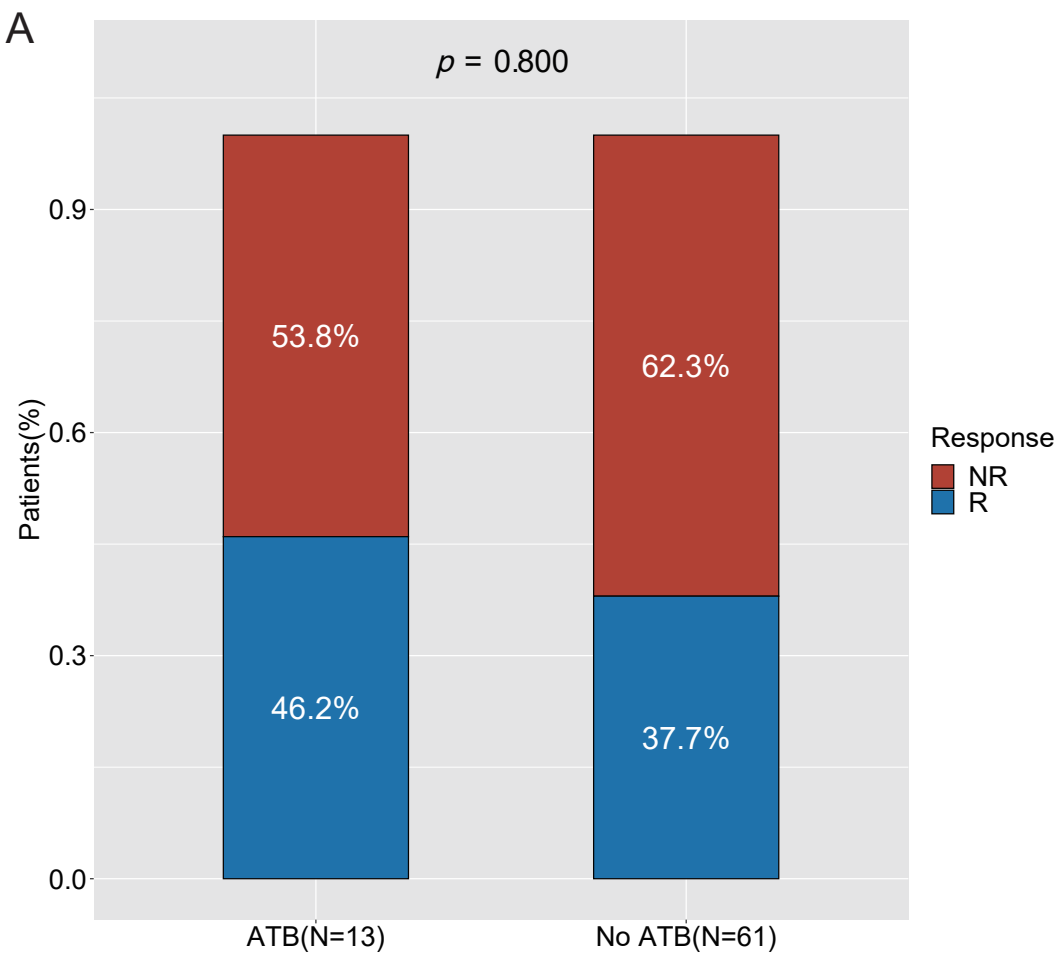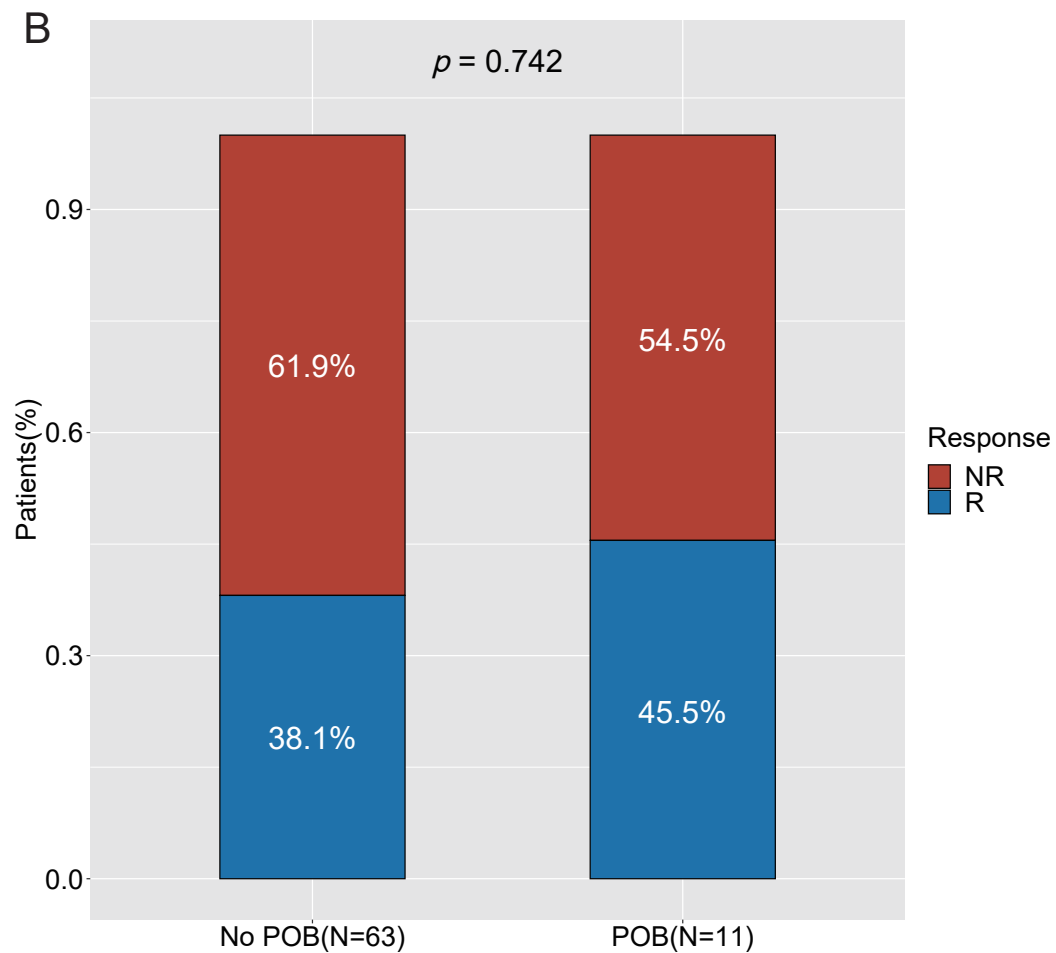

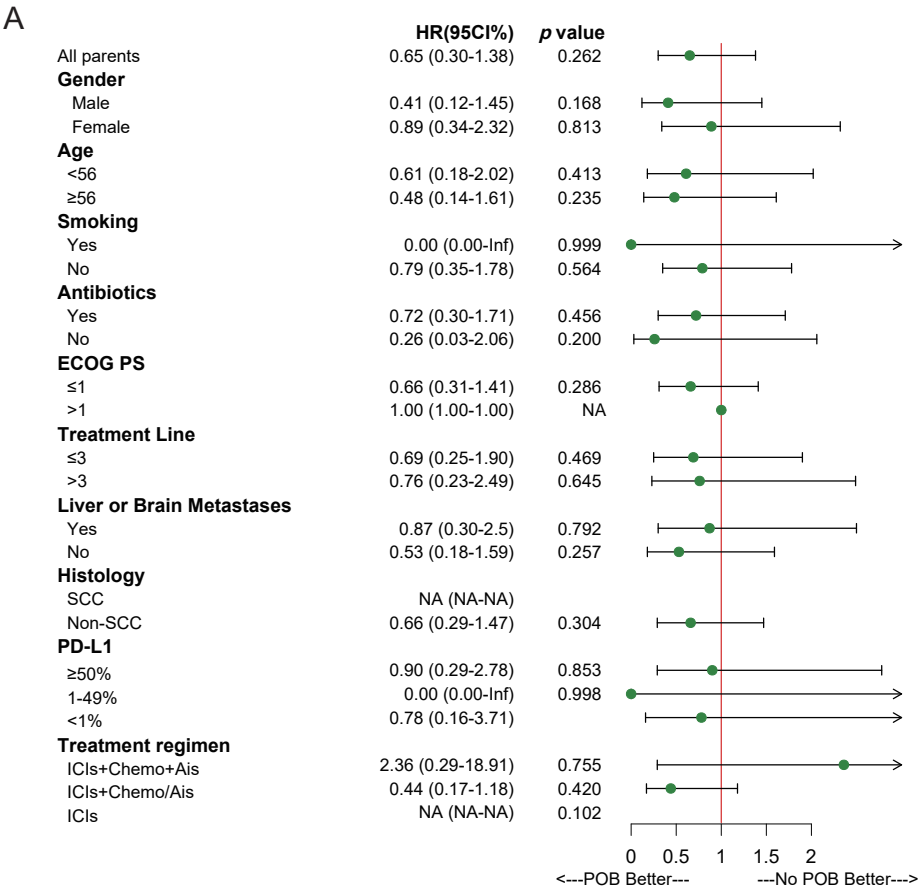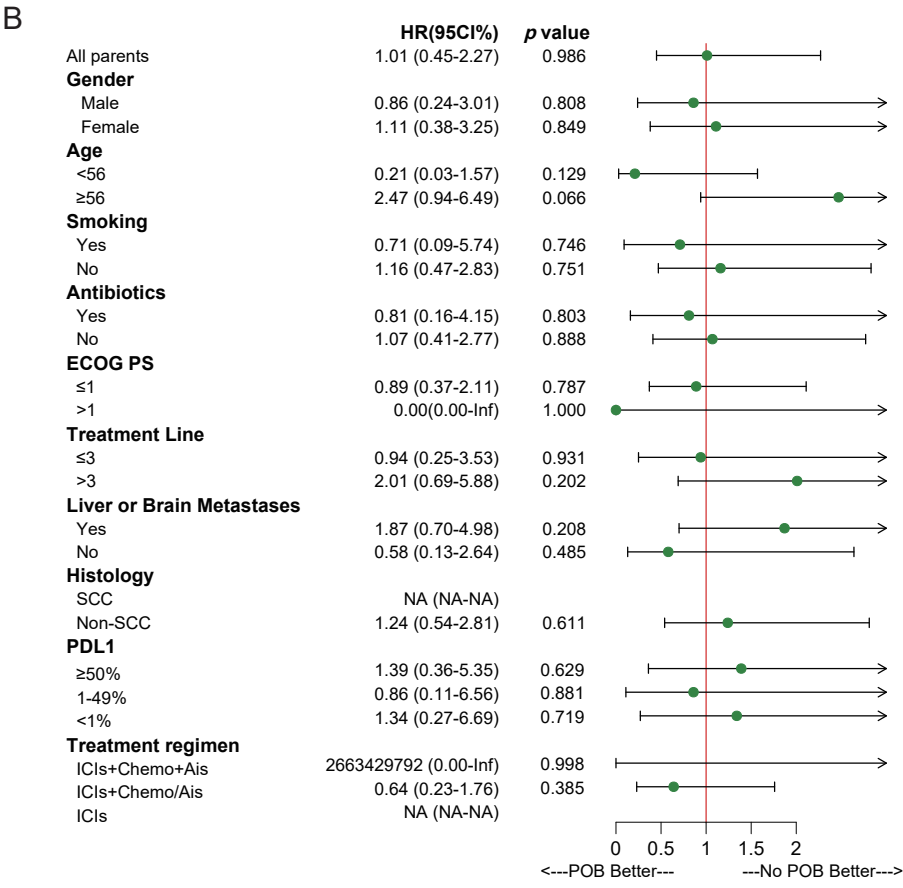

SFig4

A

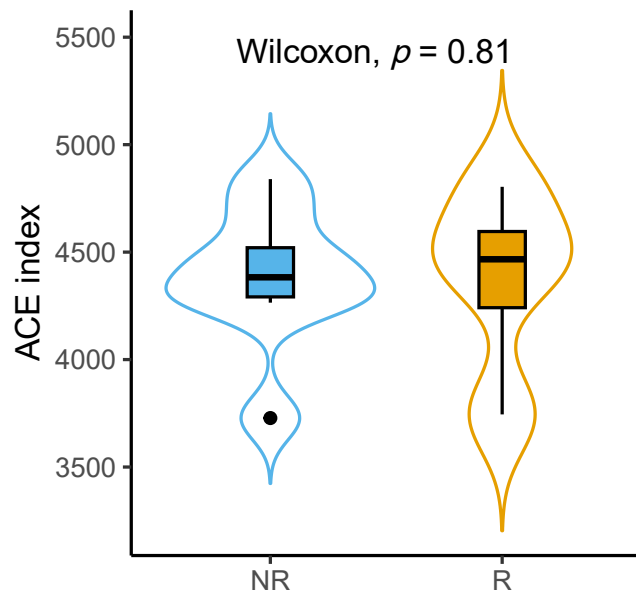

B

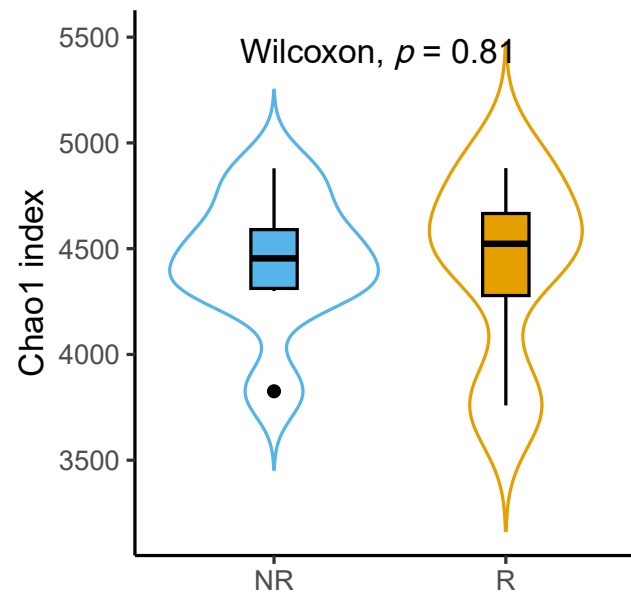

C

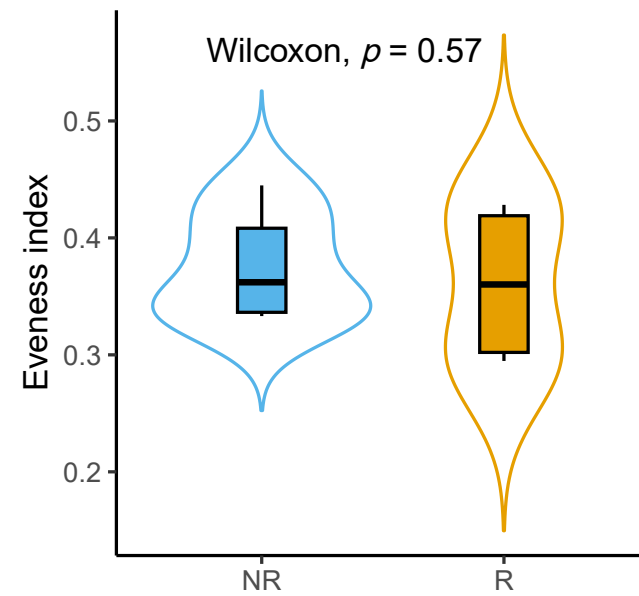

D

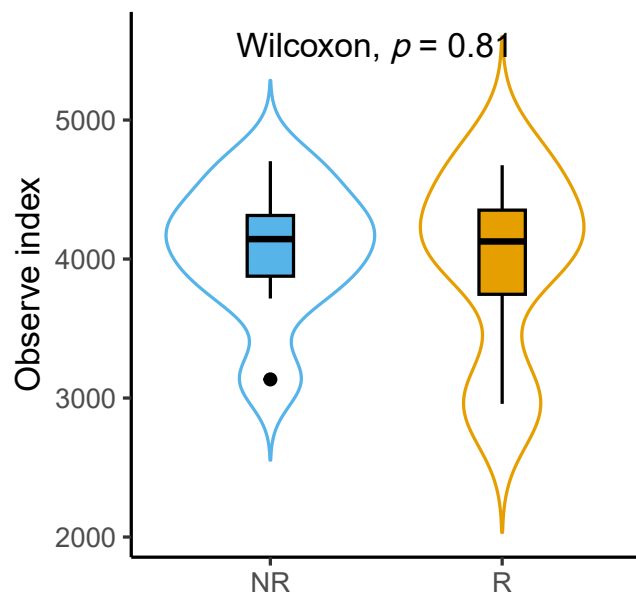

E

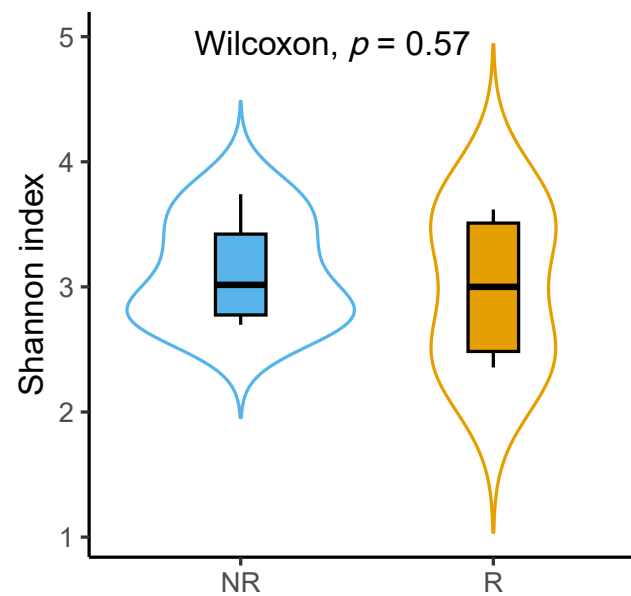

F

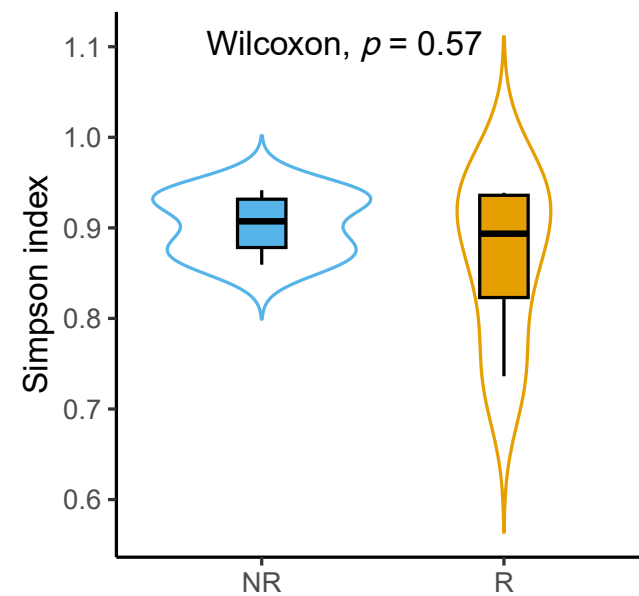

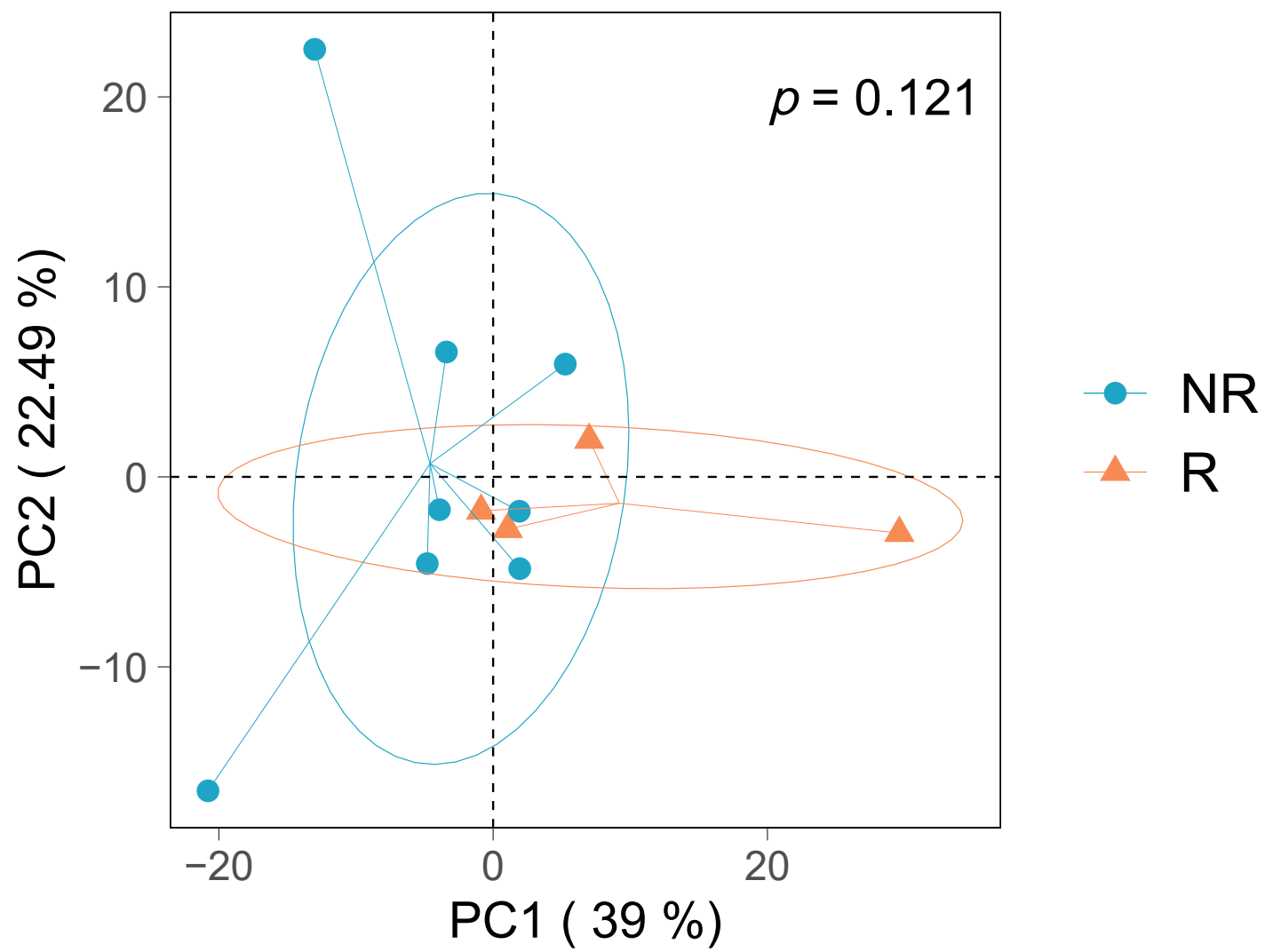

Supplement: Supplementary file 1 — Additional file 1: Figure S1. Study flowchart. Figure S2. Effects of ATB and POB treatments on ICIs responses. (A) With and without ATB treatment. (B) With and without POB treatment. ATB, antibiotic; POB, probiotic. Figure S3. Forest plot of subgroup analysis according to baseline characteristics for progression-free survival (PFS) (A) and overall survival (OS) (B) in all included patients. ECOG PS, Eastern Cooperative Oncology Group performance status; SCC, squamous cell carcinoma; ICIs, immune checkpoint inhibitors; Chemo, chemotherapy; Ais, Anti-angiogenesis therapy; NA, non applicable; Inf, infinite; POB, probiotic. Figure S4. Comparison of gut microbiota alpha diversity between R and NR. R, responder; NR, non-responders. Figure S5. Comparison of gut microbiota beta diversity between R and NR. R, responder; NR, non-responders. [file 12967_2024_5135_MOESM1_ESM.pdf]
